# Prevalence of positional skull deformities in 530 premature infants with a corrected age of up to 6 months: a multicenter study

**DOI:** 10.1186/s12887-019-1864-1

**Published:** 2019-12-30

**Authors:** Wang Yang, Jianping Chen, Wenzhi Shen, Chengju Wang, Zhifeng Wu, Qing Chang, Wenzao Li, Kuilin Lv, Qiuming Pan, Hongxia Li, Duyao Ha, Yuping Zhang

**Affiliations:** 1 Department of Pediatrics, the Second Affiliate Hospital of Army Medical University, No. 83 Xinqiao Street, Chongqing, 400037 China; 2 Department of Child Health Care, Yongchuan Maternal and Child Health Care Hospital of Chongqing, Chongqing, 402160 China; 3 Department of Child Health Care, Wanzhou Maternal and Child Health Care Hospital of Chongqing, Chongqing, 404000 China

**Keywords:** Brachycephaly, Plagiocephaly, Dolichocephaly, Positional deformities, Premature infants

## Abstract

**Background:**

Positional deformities (PD) are common during early infancy. Severe cases may result in facial abnormalities and be associated with delayed neurological development in infants. The earlier the detection of PD, the better the intervention effect and the lower the cost of treatment. Currently, there are many studies on PD in Europe and the United States. However, in China, there is little data on the basic metrics and incidence of PD. Premature infants have a high risk of PD. However, there are few studies on PD in premature infants globally, and none in Asia. This study aimed to investigate PD and its characteristics inpremature infants to help its early detection and intervention and thus improve the quality of life for premature infants.

**Methods:**

We analyzed 530 preterm infants who visited the outpatient departments at Xinqiao Hospital of Army Medical University and Maternal and Child Health Care Hospitals of Wanzhou and Yongchuan Districts in Chongqing from September 1, 2016, to August 31, 2017. The head shape data measured by a simple manual method were recorded. The diagonal difference (DD) between the transcranial diagonals and the cranial index (CI) was calculated. PD and its incidences indifferent gestational ages and corrected age groups were analyzed.

**Results:**

According to previously defined international diagnostic criteria, the incidence of plagiocephaly, brachycephaly, and dolichocephaly were 51.1, 85.1, and 3.0% respectively, and those of right and left plagiocephalywere69.4 and 30.6%, respectively. The incidence of PD was highest among infants with a gestational age of < 32 weeks and decreased as the gestational age increased. As the corrected age (CA) increased, the incidence of plagiocephaly and dolichocephaly decreased, and the incidence of brachycephaly increased.

**Conclusions:**

PD incidence is high among preterm infants. As gestational age decreased, PD incidence and severity increased. Therefore, healthcare providers should implement early PD detection and intervention to prevent the adverse outcomes. The extremely high incidence of brachycephaly and extremely low incidence of dolichocephaly in this study are likely to be due to the variance of cranial metrics caused by cultural differences. The Chinese standards for infant cranial measurements must be established.

## Background

Positional skulldeformities (PD) are characterized by asymmetry and flattening of the skull due to external forces [1] and are among the most commonly encountered problems during child growth and development, especially ininfants under 6 months old. PD can be classified into the following three types: (1) plagiocephaly wherein the skull is subjected to asymmetrical forces, resulting in one side of the skull becoming oblique with an increased diagonal difference (DD) between the transcranial diagonals; (2) brachycephaly refers to an excessive cranial width due to an abnormal cranial length and increased width/length ratio; and (3) dolichocephaly, which occurs on the left and right sides of the skull, is characterized by a long and narrow head shape due to the cranial anteroposterior distance being significantly longer than the left-right distance. Dolichocephaly is often detected in infants who sleep in the lateral position for long durations. In addition, there is a special type of dolichocephaly, called scaphocephaly, which is often caused by craniosynostosis or congenital maldevelopment of the brain. Its worldwide incidence rate is extremely low, approximately 3:100,000, which requires a comprehensive diagnosis combined with computed tomography (CT) and other examinations. So this condition is not usually diagnosed in primary care settings [2].

PD may cause a shifting of the eyes and ears, or one-sided forehead protrusions, resulting in facial asymmetry. Such facial changes may persist, often leading to ridicule or bullying of children, thus negatively affecting their psychosocial status. Children with severe PD may also have ear- nose-throat dysfunction (ENTD) [3] or an associated impairment in nervous system development, causing serious harm to infants. Some studies reported a correlation between the severity of plagiocephaly and the Bayley score [4] and suggested that PD can be used as an indicator of developmental retardation [5].

Current clinical studies have shown that most mild to moderate PD can be recovered to normal or be significantly corrected by sleep position correction at the early stages in infants (within 4 months of birth) [2]. The earlier PD is detected, the better the correction effect and the lower the cost. With an increase in an infant’s age, the hardness of the skull increases, the range of head movement increases and is difficult to control, and the difficulty of correction will be greater. For moderate and severe PD after 4 months of age, the child may even need to wear a helmet or undergo corrective surgery, which will greatly increase the cost of treatment, pain (for the child) and burden on the family [2]. Therefore, it is very important to detect and correct PD in early infancy.

The infantile head shape began to attract attention more than 30 years ago in developed countries, such as the United States and Canada [6, 7]. Based on extensive studies, Rossi et al. established the international criteria for PD by utilizing biomathematics and medical statistics in 1976 [8]. Subsequently, some studies proposed diagnostic criteria for PD classification [9–12]. In comparison, very few studies have been conducted in Asia. In 2005, the American scholar, Jone Stockman published a report on cranial measurements in Japan and Korea [13], but only few subsequent studies in Asia have been conducted. The studies on PD are scarce in China; There was only one previous report on physiotherapy [14] before our research on the head shape of Chinese term babies was reported [15]. Understanding the measurement data of head shape and the incidence of PD in Chinese infants is a necessary basis for early detection and correction of PD in Chinese infants.

In the field of modern neonatal medicine, medical professionals are most concerned about premature infants. Due to the immature organ function, premature infants always have some problems in the early stages of their lives, which may even affect their whole life. Preterm infants are considered a high-risk population due to their preterm birth, soft skull, and prolonged supine sleeping position compared with their full-term counterparts. Persistent PD may affect the appearance of premature babies and even pose a threat to their neurological development. However, because premature babies are born early, if PD is detected early, they have more time for correction than full-term infants. Therefore, in order to prevent and treat PD in premature infants, it is critical to understand the incidence and regularity of PD in premature infants. Therefore, we evaluated the incidence and characteristics of PD by conducting skull measurements in 530 preterm infants from three medical institutions located in central, eastern, and western Chongqing.

## Methods

### Research subjects

In this study, we enrolled 683 premature infants of up to 6 months corrected age (CA) who visited the outpatient department for the first time of the primary care clinic of Xinqiao Hospital of Army Medical University, Maternal and Child Health Care Hospitals of Wanzhou and Yongchuan Districts in Chongqing from September 1, 2016, to August 31, 2017. Data were collected once for each infant. After screening according to selection criteria, 530 patients were finally included. Among the 153 excluded infants, 62 were twins, 3 were triplets, 39 were small for their gestational age, 10 were diagnosed with HIE in the neonatal period, 17 were diagnosed with global developmental delay, and 22 were diagnosed with PD in other hospitals and referred to our hospital.

The inclusion criteria were as follows: (1) gestational age at birth < 37 weeks. (2) single birth. (3) appropriate for gestational age. We excluded (1) patients diagnosed with brain injury, dysplasia or global developmental delay within 6 months, (2) infants with congenital muscular torticollis, (3) infants whose PD was diagnosed in other hospitals and referred to the study centers, and (4) infants with cranial abnormalities due to definite craniosynostosis.

### Methods of measurement

Measurement was performed according to the HeadsUpTM technique described in other studies [16–18]. Before the test, the survey personnel were intensively trained. Through the reliability test, the measurement difference of all survey personnel was less than 5%; each parameter was measured thrice in a patient, and the mean value was considered for analysis.

**Fig. 1:**
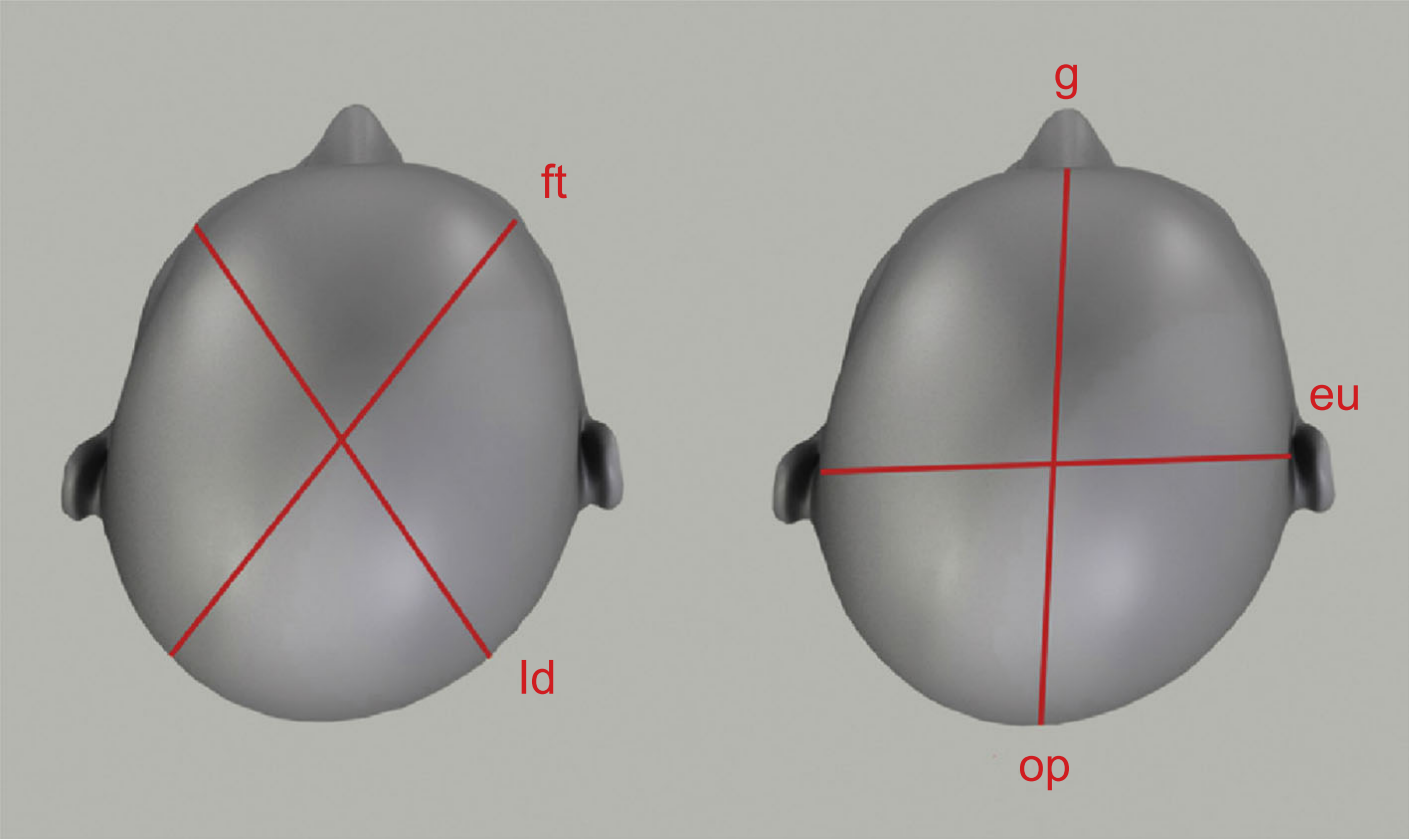
Anthropometric landmarks for measurement, ft., frontotemporale point; ld, lambdoid point; g, glabella; op, opisthocranion; eu, eurion

With the infant facing the examiner, the examiner held the infant’s head in the center. According to Wilbrand’s standardized scheme [19], we proceeded as follows: Measurements were performed according to a standard protocol determined previously [20]. The following parameters were obtained: cranial circumference, cranial length (glabella[g]–opisthocranion[op]), cranial width (eurion [eu]–eurion [eu]), and transcranial diagonals A and B (frontotemporale[ft]–lambdoid[ld]) (Fig. 1).

The following derivatives were calculated based on the measurements. Diagonal difference (DD) between the transcranial diagonals: difference in the bilateral cranial oblique dimension (mm); cranial index (CI): cranial width/cranial length × 100%.

To identify the CI, DD, and incidence of PD in preterm infants with different gestational age at birth, all preterm infants were divided into the following three groups based on the gestational age at birth: < 32 week, 32–34 week, and > 34-week groups. Moreover, to determine the incidence and severity of PD in preterm infants with different CA, the infants were divided into the following four groups based on the CA: CA < 0, 0 ≤ CA ≤ 2 months, 3 ≤ CA ≤ 4 months, and 5 ≤ CA ≤ 6 months.

### Diagnostic criteria

Diagnostic criteria were based on those specified in the *Handbook of Physical Measurements* and those adopted in the study by Looman and Wilbrand [9, 19, 21]. The diagnostic criteria for plagiocephaly were as follows: abnormal, DD ≥ 3 mm; mild, 3 mm ≤ DD < 10 mm; moderate, 10 mm ≤ DD < 12 mm; and severe, DD ≥ 12 mm. The diagnostic criteria for brachycephaly were as follows: abnormal, CI ≥ 82%; mild, 82% ≤ CI < 90%; moderate, 90% ≤ CI < 100%; and severe, CI ≥ 100%. The diagnostic criteria for dolichocephaly were as follows: abnormal, CI < 76%; mild, 76% > CI ≥ 74%; moderate, 74% > CI ≥ 70%; and severe, CI < 70% [16].

### Statistical analysis

Statistical analysis was performed using the IBM SPSS Statistics version 22.0 for Windows (IBM Germany GmbH, Ehningen, Germany). Measurement data are expressed as mean ± standard deviation or median ± standard deviation. Student’s t-test was used for comparisons between different groups of preterm infants with plagiocephaly and brachycephaly. *P* < 0.05 was considered statistically significant.

## Results

### Patient characteristics

The gestational age, birth weight, gender and delivery type of the 530 premature infants included are shown in Table 1. There was no statistical difference between the groups in terms of gender and delivery type (Table 1).

**Table 1:** Patient characteristics

	<32	32~34	34~37	
n	137	214	179	
gestational age (week)	28.4 ± 1.7	33.1 ± 0.8	35.6 ± 1.2	
birth weight (g)	1328 ± 260	2129 ± 159	2571 ± 168	*F = 15.694, P<0.001*
gender (Male/Female)	67/70	102/112	83/96	*χ*^*2*^ = 0.202, *P* = 0.904
Delivery type (natural birth/cesarean section)	79/58	116/98	94/85	*χ*^*2*^ = 0.845, *P* = 0.655

### Incidence and severity of different types of PD

**Fig. 2:**
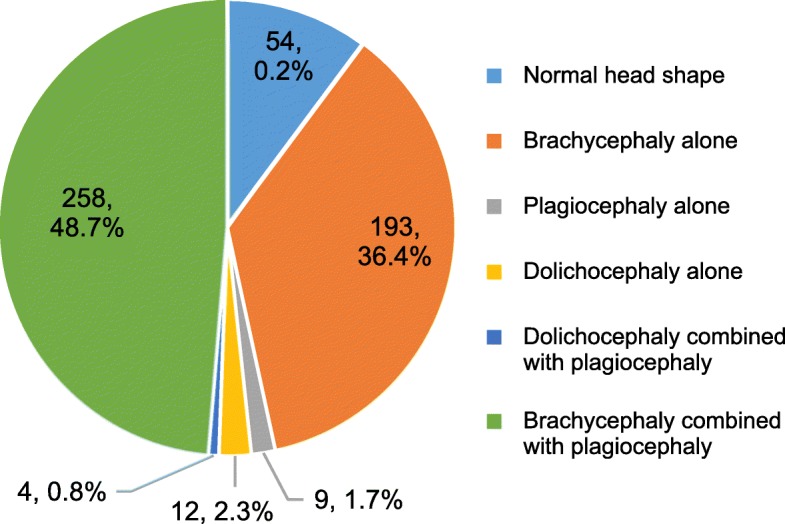
Incidence and severity of different types of PD, C:Severity of dolichocephaly in preterm infants with different gestational ages at birth

Among 530 premature infants, only 54 (10.2%) had normal cranial shape. There were 258 cases with brachycephaly combined with plagiocephaly, accounting for the highest proportion (48.7%). Next, 193 cases (36.4%) had brachycephaly alone. Only 9 cases (1.7%) had plagiocephaly alone and 12 cases (2.3%) had dolichocephaly alone. The smallest number of babies had dolichocephaly combined with plagiocephaly (0.8%) (Fig. 2).

A total of 271 cases met the diagnostic criteria for plagiocephaly (51.1%) among the 530 preterm infants. Among all the infants with plagiocephaly, 246 (90.8%) cases were mild, 15 (5.5%) were moderate, and 10 (3.7%) were severe.

A total of 451 cases met the diagnostic criteria for brachycephaly (85.1%) among the preterm infants. Among all the infants with brachycephaly, 242 (53.7%) cases were mild, 181 (40.1%) were moderate, and 28 (6.2%) were severe.

A total of 16 cases met the diagnostic criteria for dolichocephaly (3.0%) among the preterm infants. Among all the dolichocephaly cases in our study, 12 (75.0%) cases were mild, 4 (25.0%) were moderate, and none were severe (Table 2).

**Table 2:** Incidence and severity of plagiocephaly and brachycephaly in preterm infants

	Mild	Moderate	Severe	Total
Plagiocephaly				
DD value	0.537 ± 0.192	1.030 ± 0.047	1.384 ± 0.160	
N	246	15	10	271
Incidence rate	46.4%	2.8%	1.9%	51.1%
Proportion	90.8%	5.5%	3.7%	100%
Brachycephaly				
CI value	0.860 ± 0.022	0.934 ± 0.026	1.048 ± 0.092	
N	242	181	28	451
Incidence rate	45.7%	34.1%	5.3%	85.1%
Proportion	53.7%	40.1%	6.2%	100%
Dolichocephaly				
CI value	0.752 ± 0.006	0.728 ± 0.005	–	
N	12	4	0	16
Incidence rate	2.3%	0.7%	0	3.0%
Proportion	75.0%	25.0%	0	100%

CI, cranial index; DD, diagonal difference; N, number of cases

### Comparison of the incidence of left- and right-sided plagiocephaly

Among the 271 cases of plagiocephaly, 188 (69.4%) cases met the right-sided plagiocephaly criteria (right oblique dimension – left oblique dimension ≥0.3 cm), which was significantly higher than the number of cases with left-sided plagiocephaly [83 (30.6%)] (*P* < 0.05).

### CI, DD, and incidence of PD in preterm infants with different gestational ages at birth

DD (0.516 ± 0.363) and CI (0.885 ± 0.060) of the < 32-week group were significantly higher than those of the other two groups (*p* < 0.05). DD (0.437 ± 0.346) and CI (0.882 ± 0.067) of the 32–34-week group were significantly higher than those of the > 34-week group (0.387 ± 0.305, 0.875 ± 0.066). The infants in the < 32- week group had the highest incidence of plagiocephaly (58.4%) and brachycephaly (92.6%), which was remarkably higher than that of the other two groups (p < 0.05). The incidence of plagiocephaly (49.1%) and brachycephaly (83.7%) in the 32–34 week group was slightly higher than that of the > 34-week group (p < 0.05). The < 32-week group had the lowest incidence of dolichocephaly (1.5%), and the 32–34-week group (3.7%) and > 34-week groups (3.4%) showed similar incidence rates (Table 3).

**Table 3:** CI, DD, and incidence of PD in preterm infants with different gestational ages at birth

Group	N	Plagiocephaly		Brachycephaly		Dolichocephaly		DD	CI
		N	%	N	%	N	%		
W < 32 weeks	137	80	58.4%	129	92.6%	2	1.5%	0.516 ± 0.363	0.885 ± 0.060
32w ≤ W ≤ 34w	214	105	49.1%	177	83.7%	8	3.7%	0.437 ± 0.346^a^	0.882 ± 0.067^a^
W > 34 weeks	179	86	48.0%	145	81.0%	6	3.4%	0.387 ± 0.305^ab^	0.875 ± 0.066^ab^

a: p < 0.05, compared with the < 32-week group; b: *p* < 0.05, compared with the 32–34-week group

CI, cranial index; DD, diagonal difference; N, number of cases; PD, positional deformity; W, Gestational week

### Severity of PD in preterm infants with different gestational ages at birth

**Fig. 3:**
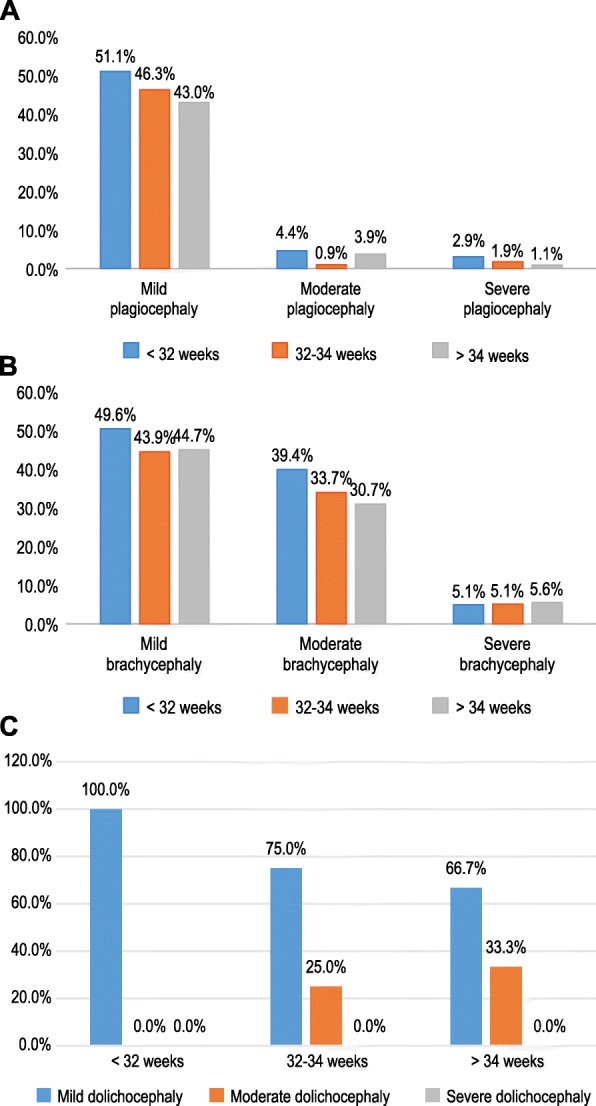
**a** Severity of plagiocephaly in preterm infants with different gestational ages at birth. **b** Severity of brachycephaly in preterm infants with different gestational ages at birth, **c** Severity of dolichocephaly in preterm infants with different gestational ages at birth

Mild plagiocephaly was most commonly observed in all three groups. The incidence of mild, moderate, and severe plagiocephaly among the very preterm infants (< 32-week group) was higher than that in the other two groups comprising infants with greater gestational ages at birth (Fig. 3a).

Mild-moderate brachycephaly was the most common in all three groups. The incidences of mild and moderate brachycephaly among the very preterm infants (< 32-week group) were higher than those in the other two groups with greater gestational age at birth, but the incidence of severe brachycephaly was similar in all three groups (Fig. 3b).

Mild-moderate dolichocephaly was the most common in all three groups. There were no case of severe dolichocephaly. There were no cases of moderate dolichocephaly in group < 32 weeks, and the proportion of moderate dolichocephaly increased with the increase of gestational age at birth. Moderate dolichocephaly in > 34 week group accounted for 33.3% of all dolichocephaly in the gestational age group (Fig. 3c).

### CI, DD, and incidence of PD in preterm infants with different CAs

As CA increased, the DD and incidence of plagiocephaly gradually decreased. The CI reached a peak at a CA of 0–2 months and subsequently decreased. However, the incidence of brachycephaly gradually increased as CA increased (Table 4).

**Table 4:** CI, DD, and incidence of PD in preterm infants with different CAs

Group	N	Plagiocephaly		Brachycephaly		Dolichocephaly		DD	CI
		N	%	N	%	N	%		
CA < 0	78	42	53.8%	58	74.4%	4	5.1%	0.492 ± 0.357	0.867 ± 0.073
0 ≤ CA ≤ 2 m	212	113	53.3%	172	81.1%	8	3.8%	0.463 ± 0.347^a^	0.937 ± 0.067^a^
3 ≤ CA ≤ 4 m	123	61	49.6%	112	91.1%	4	3.3%	0.409 ± 0.310^ab^	0.902 ± 0.066^ab^
5 ≤ CA ≤ 6 m	117	55	47.0%	109	93.2%	0	0%	0.342 ± 0.280^abc^	0.896 ± 0.057

a: *p* < 0.05, compared with the CA < 0 m group; b: *p* < 0.05, compared with the 0 ≤ CA ≤ 2 m group; c: *p* < 0.05, compared with the 3 ≤ CA ≤ 4 m group

CI, cranial index; DD, diagonal difference; CA, corrected age; N, number of cases; PD, positional deformity

## Discussion

In 1992, the American Academy of Pediatrics issued the guideline of “back to sleep,” suggesting a supine sleeping position in infancy to prevent Sudden Infant Death Syndrome (SIDS). This guideline decreased the incidence of SIDS by 40% [17]. Canada released the same consensus in 1999, reporting a decreased occurrence of SIDS from 144 cases (26% of all neonatal deaths) in 1999 to 76 cases (18% of all neonatal deaths) in 2004 [18]. However, the supine sleeping position may lead to a new problem, namely, an increased incidence of positional deformities (PD) [22, 23]. It is reported that the incidence of PD increased from 5% in the 1990s to 20–30% in 2010 [24]. In the past 10 years, research on PD has gradually increased in Western countries [25]. Several studies have suggested diagnostic criteria for PD in infants. Based on the current research data, the commonly adopted standard worldwide is based on the Handbook of physical measurements and the criteria adopted in the study of Looman WS and jan-falco Wilbrand [10, 16, 26]; we adopted the same standard in this study.

However, the differences in infant PD in different cultural backgrounds have not been fully studied. In Asia, there are relatively few studies on head shape. A report on school-age children shows that the mean CI is 82.1% (men) and 83.3% (women) in Japan [34] and 84.8% in South Korea [35]. This is different from normal CI (76–81%) in European and American countries, indicating that differences in cultural backgrounds have a greater impact on the head shape of infants. Therefore, it is important to understand the normal distribution of head shape in Chinese infants for the early diagnosis and treatment of PD. We previously conducted a study on the head shape of 3406 full-term infants in Chongqing [15], and found that according to the above international standard, the incidence of brachycephaly was up to 76%.Moreover, our study showed an average CI of 86.7% for infants at 0–6 months of term, which is significantly higher than the CI in all the countries mentioned above. The high CI is likely associated with the traditional practices of baby care in China. Since ancient times, a supine sleeping position in infancy has been advocated in the Chinese culture. Other researchers have also noticed the variance of skull metrics in different areas and cultures, because cultures who put their infants to sleep in the supine position have more brachycephalic CI than cultures who put their infants to sleep in the prone position [27–29].

Given that the skull metrics of Chinese infants are different from those of infants in Western countries, which caused the high incidence of PD in this study, the Western diagnostic criteria are not suitable. Larger-scale data must be collected to determine our own criteria. We made recommendations on diagnostic criteria for PD in local infants based on data from previous full-term studies, brachycephaly’s diagnostic criteria was CI greater than 91%, which was significantly higher than the European and American standards. However, as this study is limited to three medical institutions in southwest China and does not represent the overall situation in China, a nationwide multi-center study is needed to provide a more reliable diagnostic criteria.For the above reasons, we still adopt the internationally accepted diagnostic standard in this study. Although our findings may have overestimated brachycephaly incidence, the high incidence of PD in premature infants and the changing trend of head shape with gestational age at birth and CA observed in this study may still be of significance.

To our knowledge, this study is the first to conduct a relatively large-scale study of cranial measurements in preterm infants in China. Our findings showed that the incidences of plagiocephaly and brachycephaly among preterm infants in Chongqing were significantly higher than those among local term infants (51.1%/43, 85.1%/76%). Compared to internationally reported rates in infants, i.e., 3.9–57.2% (plagiocephaly), 16–48% (brachycephaly), and 21–88% (dolichocephaly), the incidence of dolichocephaly in preterm infants was similar to that in local full-term infants (3.2% vs 3.0%) [12, 20, 24, 30]. The incidence of plagiocephaly in premature infants in this study is high. The incidence of brachycephalyis is much higher than that previously reported, and the incidence of dolichocephaly was much lower than that previously reported. Preterm infants are born earlier than full-term babies and have a softer skull; underdeveloped systems, organs, and muscles; a high head-weight ratio; andmuscle weakness. Thus, their ability to move their head is poor, and they need a longer sleep period. With their inability to rotate the head freely or increase vertical motion, the head is pressed against the mattress for a prolonged time in such a way that the skull becomes progressively more deformed [31]. Therefore, PD in premature infants require more attention from professionals and caregivers, and early and continuous monitoring, such as weekly measurement, of the head shape of premature infants, should be encouraged. If PD are detected, early correction should be performed for obtaining good results when the skull of premature infants is not strong enough and head movement cannot be autonomously carried out.

In this study, the incidence of right-sided plagiocephaly was higher than that of left-sided plagiocephaly (69.4%/30.6%), which is consistent with results obtained in the study by Kluba et al. in full-term infants [27]. The fetus rotates in the uterus and contacts the birth canal, with the head positioned downward. In most cases, the apex of the head is located in the birth canal, and the left occipital lobe is positioned forward, causing the right occipital lobe to press against the maternal pelvis and the left forehead to be in contact with the lumbosacral vertebrae [18, 24, 28]; therefore, the right side is pressed for a long time, leading to skull deformation. This asymmetry is persistent and aggravated after birth, which may be due to the infant’s sleeping position, the way parents hold their children, and other factors. Further epidemiological studies are needed to clarify the cause of this asymmetry [32, 33].

Our study showed that infants < 32 weeks had higher DD values, CI values, and incidence of PD than infants > 32 weeks, and the incidence of plagiocephaly was reduced with the increase in gestational age, as expected. Short gestational age at birth results in a soft and more flexible skull and a poorer ability to move the head, and the head may turn to one side in the supine position due to gravity. In addition, preterm infants who are < 32 weeks require more medical interventions, such as respiratory support in an incubator. They are often placed in the supine position with their head turned to one side during the procedure. This position greatly increases the incidence of plagiocephaly. Thus, some researchers suggested that plagiocephaly should be regarded as a characteristic head shape of preterm infants [29]. The analysis of brachycephaly in preterm infants with different gestational ages at birth found that the incidence of brachycephaly is also reduced with the increase in gestational age at birth; however, the incidence is still higher than in the current report. The brachycephaly was mainly mild and moderate, which indicates that the number of cases with CI values of 82–90% is similar to that with CI values of 90–100%, suggesting that it is likely that caregivers continuously change the sleeping position of infants, thereby adjusting the CI value to 82–100%, as flatter heads are preferred by Chinese parents. The incidence of dolichocephaly is generally lower, but the incidence in preterm infants ≥32 weeks gestational age is higher than that in those < 32 weeks, which may be related to the fact that preterm infants with the younger gestational age lie supine for longer in the early development stage and the anterior and posterior diameter of the skull is shortened due to the compression of occipital bone.

Our results show that with the increase in the CA, the DD value and incidence of plagiocephaly decrease, likely because caregivers notice and correct the head shape issue and infants are gradually able to move their head; thus, different parts of the skull are compressed evenly. Regarding the brachycephaly-related indicators, we found that the CI value reaches a peak at a CA of 0–2 months (0.937 ± 0.067) and then decreases gradually with the increase in CA, which is consistent with the findings of Ifflaender et al. [16, 29]. The CI value initially increases after birth due to the pressure exerted on the infants’ heads on the mattress because the infants are not able to move their head freely. After a CA of 2 months, with the increase in the ability of the infants to move their head, the voluntary head turning activity of infants increases, which prevents further head flattening. The incidence of brachycephaly in this study did not decrease with CA, but rather gradually increased, which is not consistent with the gradual decrease in the CI value. It is obscure why the CI decreases, but the incidence of brachycephaly increases as the CA exceeds 3 months. Further analysis revealed that the high incidence in the 3–4 month and 5–6 month CA groups was based on the aforementioned diagnostic criteria, and the corresponding CI value was mainly in the range of 0.81–0.92; the head shapes corresponding in this range are acceptable to most parents. This range is similar to the revised reference value for brachycephaly suggested by Meyer-Marcotty et al. [31]. Interestingly, a German study on PD in premature infants showed that the incidence of dolichocephaly was as high as 88% when premature infants were discharged from hospital, of which 77% were moderate to severe, 30% at 3 months CA, and 21% at 6 months CA. In our study, the incidence at pre 0 months, 0 to 2 months, 3 to 4 months, and 5 to 6 months were 5.1, 3.8, 3.3, and 0%, respectively, which showed significant differences between the two studies. The reason may be that after premature birth or in the NICU, German babies are more likely to be placed in the prone position, while Chinese children are more likely to be placed in the supine position. This intriguing finding is yet another example of how cultural differences in nurturing habits can have a huge effect on the shape of the skull. These findings strongly suggest that localized infant cranial metrics should be developed to accurately identify infants who really need intervention.

In this study we adopted the manual measurement method based on Wilbrand’s standardized scheme [19]. While not as accurate as the 3-D scan, the method is simple and quick to use, making it ideal for use in situations with large numbers of patients or when repeated weekly monitoring of head shapes is required. This measurement is therefore well suited for use in primary care settings. There are several limitations to our study. The assessment of incidence was based on international standards, which may not be suitable for the needs of our in-depth research. Another limitation is the cross-sectional study design; thus, long-term follow-up of cranial changes with the development, intervention methods, and their effects in premature infants might be more informative.

## Conclusion

This study confirms that the incidence of PD in preterm infants is high, and the risk increases with younger gestational ages at birth. Considering that early intervention is important, caregivers and healthcare providers should pay attention to the head shape of the infants to facilitate early recognition and treatment of PD. Meanwhile, this study also reported that due to the different traditions regarding baby care, cranial metrics may vary in different regions and countries. To accurately identify infants who require intervention as early as possible, data from a large sample must be collected to establish head shape norm of Chinese infants.

## Data Availability

The dataset being analyzed/used during the current study is available from the corresponding author on reasonable request.

## Abbreviations

CA: Corrected age
CI: Cranial index
DD: Diagonal difference
PD: Positional deformities
